# Polarization-Insensitive Metasurface with High-Gain Large-Angle Beam Deflection

**DOI:** 10.3390/ma17235688

**Published:** 2024-11-21

**Authors:** Huanran Qiu, Liang Fang, Rui Xi, Yajie Mu, Jiaqi Han, Qiang Feng, Ying Li, Long Li, Bin Zheng

**Affiliations:** 1 Hangzhou Institute of Technology, Xidian University, Hangzhou 311231, China; 2 Key Laboratory of High-Speed Circuit Design and EMC of Ministry of Education, School of Electronic Engineering, Xidian University, Xi’an 710071, China; 3 The Research Institute for Special Structures of Aeronautical Composite AVIC, Innovation Center for Electromagnetic Functional Structure, Jinan 250023, China; 4 National Key Laboratory of Radar Signal Processing, Xidian University, Xi’an 710071, China; 5 State Key Laboratory of Extreme Photonics and Instrumentation, ZJU-Hangzhou Global Scientific and Technological Innovation Center, Zhejiang University, Hangzhou 310027, China; 6 International Joint Innovation Center, The Electromagnetics Academy at Zhejiang University, Zhejiang University, Haining 314400, China; 7 Jinhua Institute of Zhejiang University, Zhejiang University, Jinhua 321099, China

**Keywords:** metasurface, high gain, polarization insensitive, large-angle beam deflection, Fabry–Perot cavity (FPC) theory, Phase Gradient Partially Reflective Metasurface (PGPRM)

## Abstract

Metasurfaces have shown great potential in achieving low-cost and low-complexity signal enhancement and redirection. Due to the low transmission power and high attenuation issues of current high-frequency communication technology, it is necessary to explore signal redirection technology based on metasurfaces. This paper presents an innovative metasurface for indoor signal enhancement and redirection, featuring thin thickness, high gain, and wide-angle deflection. The metasurface integrates the design principles of a Fabry–Perot cavity (FPC) theory with a Phase Gradient Partially Reflective Metasurface (PGPRM). Its unit is a fishnet structure with a substrate only 1/33 λ thin. Based on the precise phase control of the dual-layer PGPRM (with an inter-layer distance of 8 mm), the proposed metasurface can obtain phase coverage as small as 78° while achieving high-gain beam deflection as large as 47°. Simulation results show that within the band 8.6–9.2 GHz (6.7%), a single-layer metasurface can deflect the beam to 29° with a maximum gain of 16.9 dBi. In addition, it is also 360° polarization-insensitive in the *xoy* plane at 9 GHz with large-angle deflection characteristic retained. Moreover, cascading PGPRM can effectively improve the beam deflection angle. After analysis, the scheme with a double-layer spacing of 8 mm was ultimately selected. Simulation results show a double-layer metasurface can deflect the beam to 47° with a maximum gain of 16.4 dBi. This design provides an efficient and cost-effective solution for large-angle beam deflection with gain enhancement for indoor wireless communication.

## 1. Introduction

In modern wireless communication systems, the dramatic increase in the number of mobile devices and the growing demand for data transmission have made indoor wireless signal coverage and enhancement particularly important [1]. To solve this challenging problem, it is necessary to adopt Millimeter Wave (mmWave) communication solutions [2,3]. However, the millimeter wave signals used in 5G applications suffer severe through-wall losses in indoor environments [4,5,6]. Traditional solutions, such as increasing the number of base stations or boosting transmission power [7], often incur complex installation processes, and may lead to environmental pollution. Therefore, finding efficient and cost-effective methods for signal enhancement and redirection has become a research hotspot in wireless communication.

In recent years, metasurface technology has emerged as a significant research direction for wireless signal enhancement, owing to its unique capabilities for manipulating electromagnetic waves [8,9]. Metasurfaces can achieve flexible control of electromagnetic waves through phase, amplitude, refractive index, and polarization [10,11,12,13,14,15,16,17,18,19]. This versatility enables potential applications of metasurfaces in wireless communication, particularly for achieving passive, high-gain signal with wide-angle coverage and directional control, offering distinct advantages.

Significant progress has been made in the research of enhancing signal gain and beam deflection over recent years. One effective approach is to utilize metamaterial with a gradient refractive index or high refractive index. This configuration allows the wave to continuously deflect as it propagates through the material, thus altering the direction of the outgoing wave [10,11]. According to Huygens’ principle, metasurfaces with negative refractive indices can also be employed to change the propagation direction of electromagnetic waves [12]. Modal synthesis represents another viable technique for generating tilted beams by combining multiple radiation modes on the aperture of the metasurface [13]. Additionally, metasurfaces designed with different operating states (transmission or reflection state) in different frequency bands can achieve high gain and beam deflection, separately [14]. Furthermore, integrating active devices allows for the management of the states of metasurface units, thereby enabling beam scanning [15,16,17]. The method of combining the Fabry–Perot resonant cavity theory and Phase Gradient Partially Reflective Metasurface to achieve high-gain beam deflection or beam convergence are adopted [18,19,20,21]. On the other hand, for applications in mobile communication and indoor communication, polarization insensitivity are indispensable factors. Most metasurfaces achieve polarization insensitivity by using fully symmetrical units and arrays [22]. Many other promising applications are also conducted creatively through phase-change materials, for example, a metasurface absorber with switchable bandwidth based on the phase-change material vanadium dioxide (VO2) [23].

Specifically, this paper employs the Fabry–Perot cavity (FPC) theory to enhance the gain and achieve polarization-insensitive characteristic based on a completely symmetrical unit structure. The antenna comprises a partially reflective surface (PRS) and a feeding antenna. This structure facilitates multiple reflections of electric field radiation between the PRS and the ground plane, resulting in constructive interference with the outgoing radiation wavefront, and thus effectively increases the gain of the antenna [24,25]. The optimal conditions for achieving high gain based on FPC theory at large-angle deflection are summarized in references [26,27].

Since Capasso et al. introduced the concept of abrupt phase shifts in Fermat’s principle in 2011 and derived the generalized Snell’s law [28], the Phase Gradient Partially Reflective Metasurface (PGPRM) has emerged as an effective method for achieving beam deflection by adjusting the phase distribution of the radiation aperture. This paper designs a Phase Gradient Partially Reflective Metasurface (PGPRM), which generates tilted main beams by adjusting radiation wavefront [18,29,30,31], thereby realizing beam deflection.

In this paper, we design a high-gain beam deflection antenna based on a PGPRM integrated with an FPC structure for indoor signal enhancement and redirection. By designing the metasurface with a fishnet structure, flexible control of antenna beam direction is enabled through phase manipulation across the metasurface aperture. Polarization sensitivity analysis shows that the metasurface exhibits consistent reflection characteristic and good isolation under 360° polarization directions, confirming the polarization-insensitive property. Consequently, the direction of beam deflection in the *xoy* plane remains aligned with the rotation direction of the PGPRM.

The primary contribution of this research lies in the performance and structure of the PGPRM, achieving high gain while demonstrating significant flexibility in beam direction control. It is believed that this simple, thin, passive, and efficient metasurface holds great potential for future wireless communication systems.

## 2. Design of PGPRM

**Figure 1:**
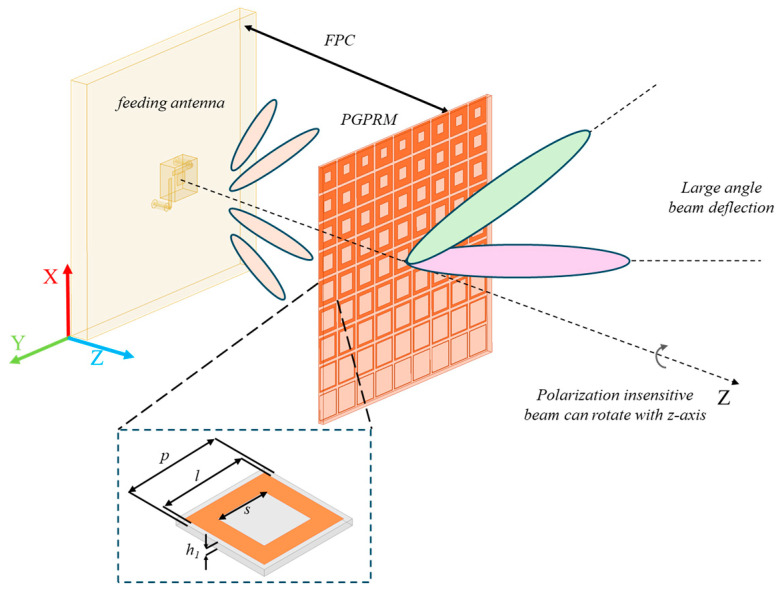
Schematic diagram of the proposed PGPRM based on FPC theory.

**Figure 2:**
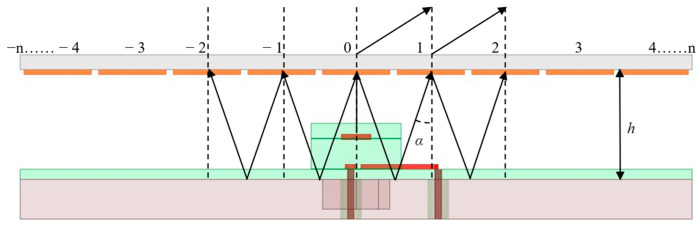
The schematic diagram of multiple reflections and deflection of electromagnetic waves.

This paper presents a PGPRM antenna based on the FPC theoretical model. The schematic diagram of the PGPRM antenna is shown in Figure 1. The electromagnetic waves radiated by the antenna undergo constructive interference within the Fabry–Perot cavity and are then deflected by the PGPRM upon emission. The schematic diagram of multiple reflections and deflection of electromagnetic waves is shown in Figure 2. The numbers in the figure are unit labels, where h represents the loading height of PGPRM. Under ideal conditions, electromagnetic waves are radiated from a radiation source, reflected infinitely in FPC, and then emitted through PGPRM. The electric field strength of the emitted electromagnetic waves is the superposition of multiple reflected electromagnetic waves [27]:

(1) $$E=\sum_{n=0}^{\infty }f\left(\alpha \right)E_{0}p^{n}\sqrt{1-p^{2}}e^{j\theta_{n}},$$

Among them,

(2) $$\theta_{n}=n\varphi =n(-\frac{4\pi }{\lambda }h\cos\alpha +\varphi_{PGPRM}+\varphi_{GND}),$$

In Equations (1) and (2), $f\left(\alpha \right)$ is the directional function of the field strength corresponding to the electromagnetic wave incident at angle α, $\theta_{n}$ is phase difference in the n th radiated ray compared to the 0th ray, p is the reflection amplitude of PGPRM, and Simplified Equation (1) yields the following:

(3) $$\left|E\right|=\left|E_{0}\right|f\left(\alpha \right)\sqrt{\frac{1-p^{2}}{1+p^{2}-2p\cos\phi }},$$

The energy density corresponding to the incident wave at angle α is as follows:

(4) $$S=\frac{1-p^{2}}{1+p^{2}-2p\cos\left(\varphi_{PGPRM}+\varphi_{GND}-\frac{4\pi }{\lambda }h\cos\alpha \right)}f^{2}\left(\alpha \right),$$

In Equation (4), where λ denotes wavelength at working frequency, the reflection coefficient of the PGPRM is $\varphi_{PGPRM}$, and the reflection coefficient of the ground plane is $\varphi_{GND}$. According to Equations (1)–(4), the energy density of the cavity at angle α is the highest when the following resonance conditions are met:

(5) $$h\cos\alpha =\frac{\varphi_{PGPRM}+\varphi_{GND}}{4\pi }\lambda +\frac{\lambda }{2}\left(N-\frac{1}{2}\right),$$

Increasing the number of layers in the PGPRM can effectively enhance the beam deflection angle. But as the number of cascaded layers increases, the loss also increases, which results in the gain decrease. Considering both the deflection angle and gain enhancement, a cascaded double-layer model is selected. Meanwhile, the PGPRM are insensitive to polarization directions, and the beam can be rotated synchronously by rotating the PGPRM around the *z*-axis.

The PGPRM unit is designed as a metal square ring. The period of the PGPRM unit should lie between 1/5 and 1/2 λ at the working frequency 9 GHz, and the period of the unit is optimized as *p* = 15 mm, which is approximately 0.45 λ [32]. The size of the metal square ring is a key factor affecting the unit’s reflection phase. Thus, selecting a larger outer side length of *l* = 13.5 mm for the metal square ring provides ample room for variation. To avoid extreme cases, a moderate initial inner side length of the metal square ring, *s* = 8 mm, is selected. The thickness of the metasurface unit is set as 1 mm, approximately 1/33 λ, which demonstrates the thin and lightweight characteristic of the PGPRM. The F4B dielectric plate exhibits excellent electrical performance of low loss and stable dielectric constant, particularly in high-frequency applications. F4B material with a dielectric constant of 2.65 is thus utilized as the PGPRM unit [33,34].

**Figure 3:**
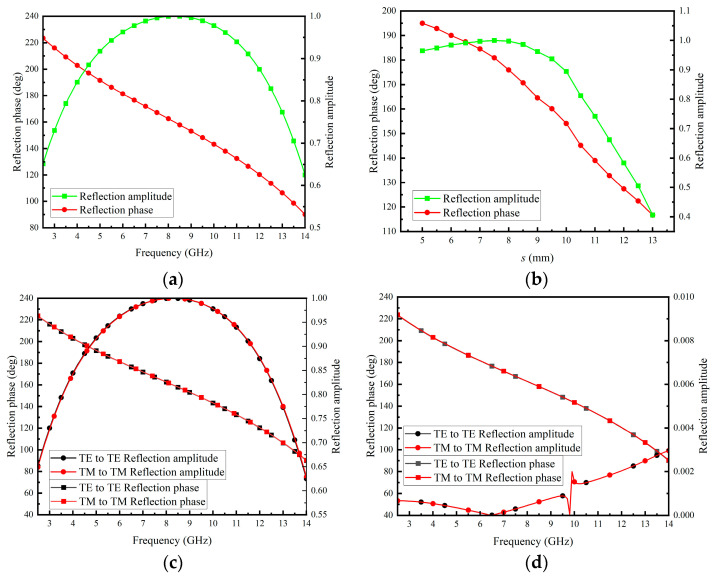
Infinite periodic model simulation of PGPRM. (**a**) Reflection phase and amplitude varying with frequency; (**b**) reflection phase and amplitude varying with the side length of the square aperture at 9 GHz; (**c**) polarization sensitivity analysis; (**d**) cross isolation analysis.

The infinite periodic PGPRM model was simulated in HFSS using the Floquet port. The Floquet port, primarily used for periodic structures, also simplifies boundary conditions with oblique incident wave setup and analysis in non-periodic structures [35]. The model’s reflection characteristic were simulated and analyzed, with the results presented in Figure 3a. The reflection phase of the model decreased with increasing frequency, while the reflection amplitude remained at a high level (>0.6). The center frequency of the PGPRM unit was set at 9 GHz. The influence of the square aperture’s side length *s* on the reflection characteristic was analyzed, with the results shown in Figure 3b. As the structural parameter *s* increased, the reflection phase decreased approximately linearly, while the reflection amplitude first increased and then decreased. Thus, the phase gradient between units can be adjusted by varying the parameter *s* of the PGPRM. Finally, the unit’s polarization characteristic was analyzed. The reflection characteristic and cross-polarization isolation of the model under TE and TM mode states were analyzed separately. The results are presented in Figure 3c,d. The results indicate that the model exhibits polarization insensitivity characteristic and extremely low cross-polarization isolation.

Based on the above analysis, metasurface units 1–9 with a gradient phase of approximately 10° can be achieved. The structural parameters of the units were [*s*_1,_ *s*_2,_ *s*_3,_ *s*_4,_ *s*_5,_ *s*_6,_ *s*_7,_ *s*_8,_ *s*_9_] = [13 mm, 12 mm, 11 mm, 10 mm, 9 mm, 8 mm, 7 mm, 6 mm, 5 mm], while the remaining structural parameters were the same with [*h*_1_, *p*, *l*] = [1 mm, 15 mm, 13.5 mm]. As the parameter *s* decreased, the units’ reflection phase increased linearly while maintaining an average phase difference of approximately 10° between adjacent units. As can be observed from Figure 3b, it is evident that the PGPRM can achieve a reflection phase coverage of approximately 78° at 9 GHz.

## 3. Gain and Beam Deflection Analysis

### 3.1. PGPRM Antenna Design

**Figure 4:**
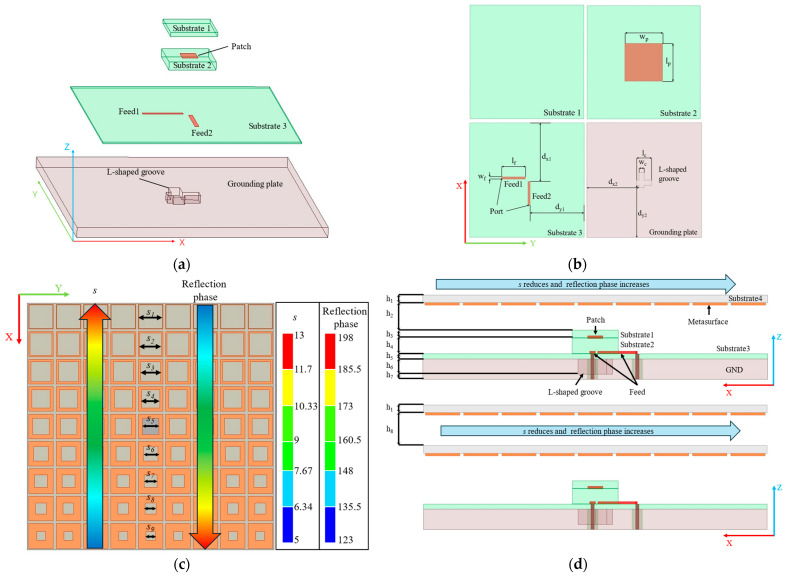
Overall schematic diagram of PGPRM antenna. (**a**) Schematic diagram of *L*-shaped groove patch antenna; (**b**) schematic diagram of each layer of *L*-shaped groove patch antenna; (**c**) top view of PGPRM antenna; (**d**) side view of single-layer and double-layer PGPRM antenna.

Feeding antennas are critical components in antenna systems. Thus, this paper employs a compact patch antenna with a relatively wide bandwidth [36]. This antenna features dual polarization, allowing the polarization mode to be altered by switching the feeding port. The design of the proposed *L*-shaped groove patch antenna alongside the PGPRM antenna is illustrated in Figure 4. The structural parameters are detailed in Table 1.

The square substrate of the antenna has a side length of 135 mm. It comprises two feeding lines, three substrates, one radiation patch, and an *L*-shaped groove ground plane. The radiation patch is positioned between substrate 1 and substrate 2. Two feeding ports are arranged vertically on substrate 3. An *L*-shaped groove is etched on the ground plane beneath the antenna to enhance coupling between the feeding lines and the radiation patch, thereby improving the impedance bandwidth [37]. Additionally, substrate 1 functions as a dielectric matching layer for the patch, further expanding the antenna’s bandwidth [38]. Thick substrate 2 enhances bandwidth by increasing radiation resistance, while thin substrate 3 minimizes unwanted stray radiation from the feeding line. All three dielectric substrate layers are constructed from F4B material, which has a dielectric constant of 2.2.

When $\alpha =0,\;\varphi_{PGPRM}=\varphi_{GND}=\pi$, Equation (5) can be simplified as follows:

(6) $$h=\frac{\lambda }{2}+N\frac{\lambda }{2},$$

where the loading height $h=h_{2}+h_{3}+h_{4}+h_{5}=16.5\;mm$. It is anticipated that the antenna will achieve high gain and beam deflection effects at 9 GHz.

### 3.2. PGPRM Antenna Simulation Results

**Figure 5:**
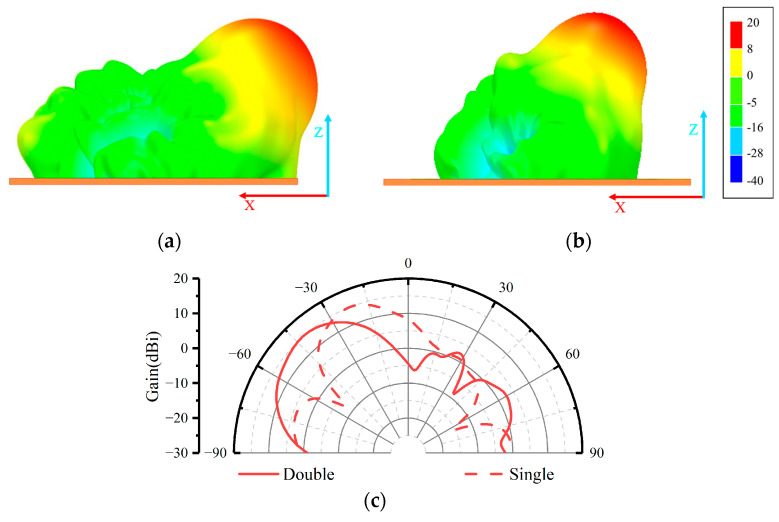
Comparison of simulation results between single-layer PGPRM antenna and double-layer PGPRM antenna. (**a**) Three-dimensional directional pattern of double-layer PGPRM antenna; (**b**) three-dimensional directional pattern of single-layer PGPRM antenna; (**c**) comparison of E-plane directional diagrams.

Employing HFSS as the simulation analysis tool, the simulation results of the PGPRM antenna were obtained. The comparison of 3D radiation patterns at 9 GHz is illustrated in Figure 5a,b, while the comparison of 2D radiation patterns is depicted in Figure 5c.

Figure 5 demonstrates that the PGPRM successfully achieved a large angle of beam deflection with significant gain improvement. The single-layer PGPRM achieved a beam deflection of 29°, whereas the double-layer PGPRM achieved a beam deflection of 47°. The maximum gain of the original feed antenna was 6.3 dBi, and the gain was effectively improved to 16.9 dBi after loading the PGPRM. The maximum gain improvement for the single-layer PGPRM antenna was 10.6 dB, while the double-layer PGPRM antenna achieved a maximum gain improvement of 10.1 dB.

**Figure 6:**
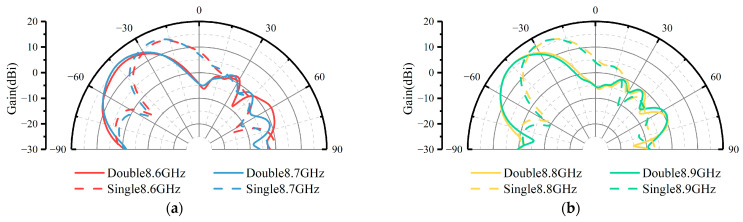

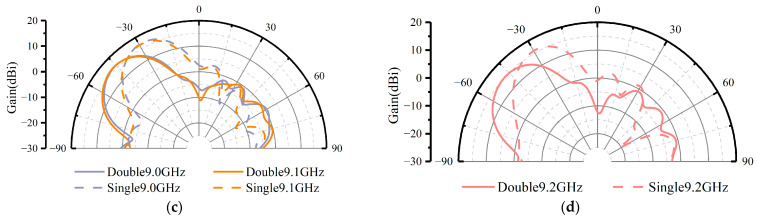
Comparison of directional patterns of PGPRM antenna in the 8.6–9.2 GHz frequency band. (**a**) At 8.6, 8.7 GHz. (**b**) At 8.8, 8.9 GHz. (**c**) At 9.0, 9.1 GHz. (**d**) At 9.2 GHz.

Further analysis of the beam deflection bandwidth of the PGPRM antenna is illustrated in the 2D radiation patterns, as depicted in Figure 6. The single-layer PGPRM achieved a beam deflection of 29° within the frequency band of 8.6–9.2 GHz, whereas the cascaded double-layer PGPRM achieved a beam deflection of 47° within the same band.

**Figure 7:**
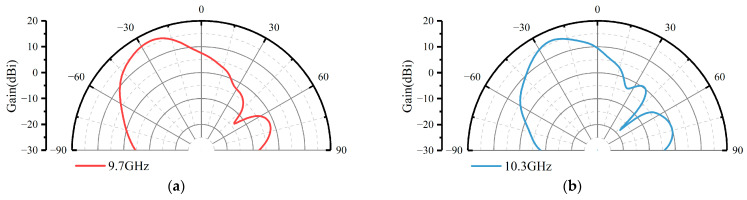

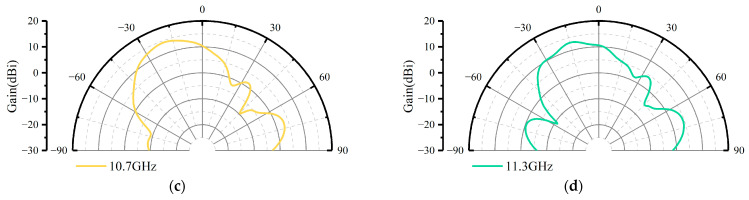
The directional patterns of single-layer PGPRM antenna at different loading heights. (**a**) At 15 mm, half wavelength of 10 GHz. (**b**) At 13.6 mm, half wavelength of 11 GHz. (**c**) At 12.5 mm, half wavelength of 12 GHz. (**d**) At 11.5 mm, half wavelength of 13 GHz.

It is noted that our proposed PGPRM unit has broadband high reflectivity characteristic. Therefore, by adjusting the loading height, its gain enhancement and beam deflection capability were tested at different working frequencies. The gain patterns of single-layer PGPRM antenna at half wavelength loading heights of 10 GHz, 11 GHz, 12 GHz, and 13 GHz are shown in Figure 7. As shown in the figure, when the loading height of PGPRM changes, the working frequency of the antenna will also change accordingly. This confirms our speculation that the broadband high reflectivity characteristic of PGPRM can be utilized to achieve gain enhancement and beam deflection in multiple working frequency.

Although PGPRM has broadband characteristic, its gain enhancement and beam deflection capability will decrease with increasing frequency due to the influence of the working bandwidth of the feed antenna. Therefore, in the future, we will improve the feed antenna to a broadband antenna to further expand the working bandwidth of PGPRM antenna.

**Figure 8:**
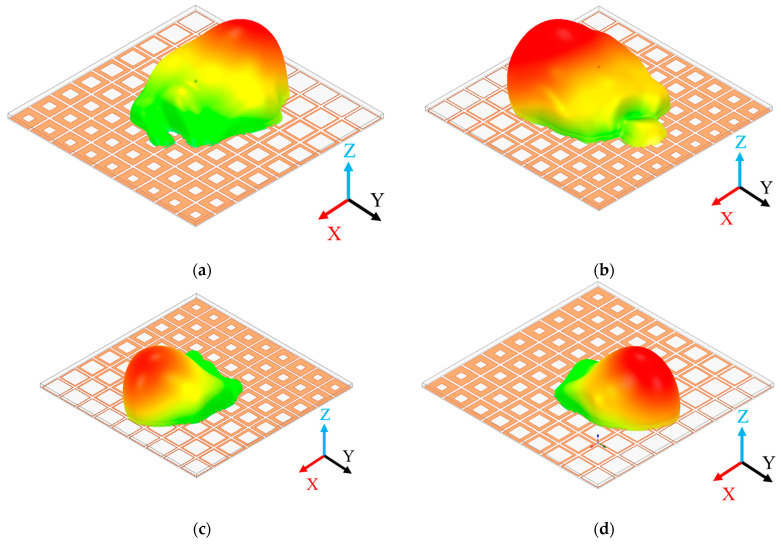
Comparison of beam directions after rotating the single-layer PGPRM around *z*-axis. (**a**) 0°; (**b**) 90°; (**c**) 180°; (**d**) 270°.

Finally, based on the polarization insensitivity of metasurface units, the PGPRM antenna should be able to achieve beam scanning within the *xoy* plane from 0 to 360° by rotating the PGPRM at 9 GHz. As shown in Figure 8, when the single-layer PGPRM rotates around the *z*-axis, the main beam follows this rotation, achieving beam scanning from 0 to 360° in the *xoy* plane. Simultaneously, the main beam consistently maintains a 29° beam deflection relative to the *z*-axis.

In summary, it can be concluded that cascading layers of PGPRM and adjusting the phase gradient enable significant beam deflection in the *xoz* plane. And thus, 360° beam scanning within the *xoy* plane can be accomplished by rotating the metasurface.

## 4. Discussion

**Figure 9:**
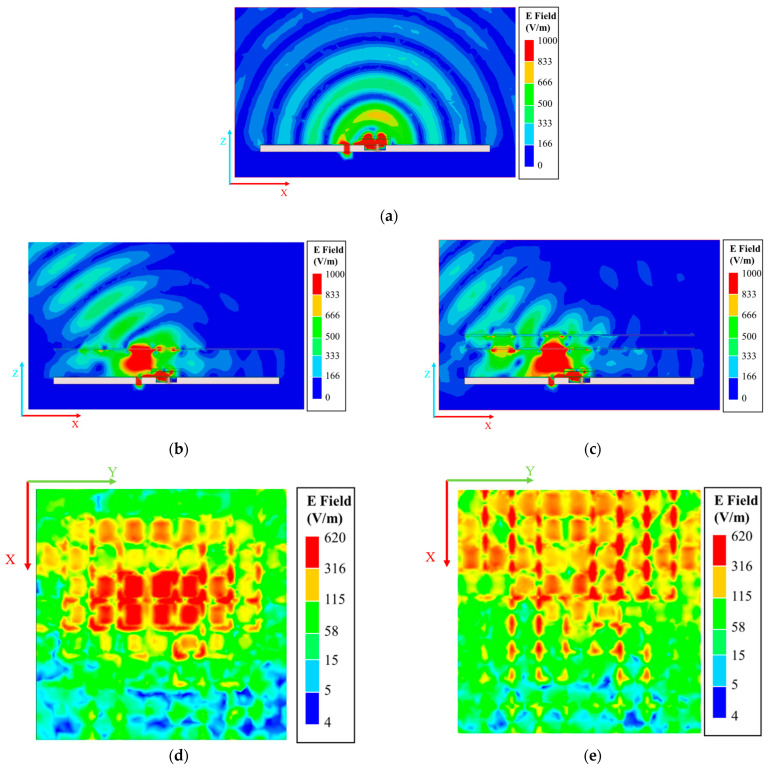
Electric field distribution before and after loading PGPRM. (**a**) Before loading PGPRM at 9 GHz; (**b**) after loading single-layer PGPRM at 9 GHz; (**c**) after loading double-layer PGPRM at 9 GHz; (**d**) surface electric field distribution of single-layer PGPRM antenna at 9 GHz; (**e**) surface electric field distribution of upper layer PGPRM in double-layer PGPRM antenna at 9 GHz.

To analyze the working mechanism of the high-gain beam deflection performance after loading the PGPRM, it is essential to examine the electric field distribution of the antenna before and after the PGPRM is loaded. Figure 9 illustrates the electric field distribution of the antenna before and after the PGPRM is loaded.

As illustrated in Figure 9, the PGPRM concentrates and deflects the emitted electric field, achieving high gain and significant beam deflection, which is consistent with prior expectations. At 9 GHz, the electric field of the PGPRM is predominantly distributed along the negative *x*-axis, corresponding to the direction of wide-angle beam deflection.

To further highlight the intrinsic advantage of the proposed design, a comparison of the overall performance for the proposed beam deflection metasurface with some representative published works is tabulated in Table 2. Reference [13] used modal synthesis to generate tilted beams and achieved high gain and large-angle beam deflection by combining two radiation modes of the metasurface. Reference [14] designed a metasurface with two working states of transmission and reflection, which can, respectively, achieve beam deflection and high gain. References [15,16,17] used a combination of active metasurface and FP cavity to generate tilted beams. By adjusting the working mode of the metasurface, high-gain beam deflection of the beam can be achieved. Reference [18] achieved high-gain beam deflection by combining an FP cavity with a Phase Gradient Partially Reflective Metasurface. Reference [19] designed a multi-beam antenna based on metasurfaces, which achieved beam deflection by loading the metasurface above the feeding network and combining four antenna elements with different orientations to achieve multi-beam functionality. Compared to references [13,14,15,16,17,18,19], this paper has lower system complexity, higher gain effect, and a wide working bandwidth.

Despite its excellent beam deflection performance and high-gain effect, the current PGPRM antenna still has the problem of high overall height during cascading, and the phase gradient of the PGPRM still needs to be improved. At the same time, the proposed PGPRM has broadband characteristic that cannot be demonstrated due to the feed antenna. In the future, we will further optimize the PGPRM antenna to reduce its height and optimize the PGPRM units’ structure to increase its phase gradient. Also, the feed antenna will be improved to a broadband antenna to broaden the working bandwidth of PGPRM antenna.

## 5. Conclusions

This paper introduces a gradient phase metasurface that effectively realizes high gain and beam deflection capabilities. The single-layer PGPRM can obtain a maximum gain of 16.9 dBi and a main beam deflection of 29° within the 8.6–9.2 GHz band (6.7%). In contrast, the double-layer PGPRM achieved a maximum gain of 16.4 dBi and a main beam deflection of 47° within the same frequency range. The proposed PGPRM has broadband characteristic, and the working frequency of the PGPRM antenna can be changed by adjusting the loading height of the PGPRM. Additionally, it demonstrates polarization insensitivity across a range of 0° to 360°. Compared to prior studies, this work demonstrates substantial gain enhancement and remarkable flexibility in controlling beam direction. These findings offer promising potential for future wireless communication systems and signal improvement in indoor communication.

## Figures and Tables

**Table 1:** Structural parameters of PGPRM antenna, unit: mm.

Symbol	Value (mm)	Symbol	Value (mm)	Symbol	Value (mm)
*s*_1_	13	*h*_1_	1	*l_c_*	12.5
*s*_2_	12	*h*_2_	11.5	*w_c_*	4.3
*s*_3_	11	*h*_3_	1.5	*l_p_*	6
*s*_4_	10	*h*_4_	3	*w_p_*	6
*s*_5_	9	*h*_5_	0.5	*d_x_*_1_	68.5
*s*_6_	8	*h*_6_	3	*d_x_*_2_	63
*s*_7_	7	*h*_7_	1	*d_y_*_1_	65.3
*s*_8_	6	*h*_8_	8	*d_y_*_2_	63
*s*_9_	5	*w_f_*	1.5	*l_f_*	15.5

**Table 2:** Comparison with previous research.

Ref.	Maximum Gain	Beam Deflection Bandwidth	Maximum Beam Deflection
[13]	7.9 dBi	5.1 GHz	35°
[14]	7.5 dBi 22.5 dBi	13.4 GHz 17.9 GHz	34° 0°
[15]	10.7 dBi	28 GHz	10°
[16]	10.5 dBi	5.44~5.66 GHz	22°
[17]	4.5 dBi	4.2~4.55 GHz	23°
[18]	12.3 dBi	9.7~10.7 GHz	58°
[19]	9.1 dBi	5.58~5.93 GHz	32°
This work	Single-layer 16.9 dBi Double-layer 16.4 dBi	8.6~9.2 GHz 8.6~9.2 GHz	29° 47°

## Data Availability

The original contributions presented in the study are included in the article, further inquiries can be directed to the corresponding authors.
